# Thyroid Function During and After COVID-19 Infection: A Review

**DOI:** 10.17925/EE.2022.18.1.58

**Published:** 2022-06-13

**Authors:** Sabri Artun Çabuk, Ayşe Zeynep Cevher, Yaşar Küçükardalı

**Affiliations:** Yeditepe University Hospital, Istanbul, Turkey

**Keywords:** Sars-coV-2, coronavirus, COVID-19, subacute thyroiditis, De Quervain thyroiditis, euthyroid sick syndrome, Hashimoto thyroiditis, Graves' disease, hypothyroidism, baricitinib

## Abstract

Severe acute respiratory syndrome coronavirus 2 (SARS-CoV-2) infection can lead to multiorgan dysfunction through pulmonary and systemic inflammation. Infection also affects the thyroid gland directly via cytopathological effects of the virus or indirectly through cytokines, complement systems and coagulation mechanisms. The thyroid gland regulates innate and adaptive immune systems by genomic and nongenomic pathways. During or after SARS-CoV-2 infection, Graves' disease and subacute thyroiditis might be triggered resulting in hyperthyroidism; alternatively, the effect of the virus on the hypophyseal.hypothalamic axis might cause central hypothyroidism. Severe cases of coronavirus disease 2019 (COVID-19) can present with hypoxia, which requires the use of dexamethasone. This can depress basal serum concentrations of 3,5,3'-triiodothyronine. Thyroid function should be monitored when using dexamethasone in patients with COVID-19. This article briefly reviews the direct and indirect effects of SARS-CoV-2 on the thyroid gland and function.

On 31 December 2019, the World Health Organization (WHO) was notified of several cases of pneumonia of unknown aetiology in Wuhan, China. After a relatively short period, officials confirmed the first case of coronavirus disease 2019 (COVID-19) reported outside of China, in Thailand, on 13 January 2020.^1^ From the start of the pandemic to 11 November 2021, there have been 251,266,207 confirmed cases of COVID-19, including 5,070,244 deaths, reported to the WHO, and the virus is still spreading.^2^

The pathogenesis of COVID-19 may affect multiple human organ systems in several ways. In particular, severe COVID-19 is characterized by organ dysfunction, hypercytokinemia and lymphopenia. The direct cytopathological damage of host cells and the dysregulated immune response caused by the severe acute respiratory syndrome coronavirus 2 (SARS-CoV-2) virus is assumed to be the primary underlying mechanisms of COVID-19.^3^

SARS-CoV-2 enters the lungs and into the lung parenchyma through the respiratory system. Eventually, spike proteins of the virus attach to angiotensin-converting enzyme 2 (ACE2), which is expressed at the surface of pneumocytes.^4,5^ ACE2 binds to the spike proteins of SARS-CoV-2 and acts as a receptor, mediating the entry of the virus to the host cells. This virus mechanism is also used to gain entry to other cell types of the human body. Many endocrine system organs have ACE2-expressing cells, such as the pancreas, testis, ovary, adrenal gland, pituitary gland and thyroid gland.^6,7^ The testis has the highest level of ACE2 expression, followed by the thyroid, whereas the hypothalamus has the lowest level.^6^

The pituitary–thyroid axis should be regarded as a vulnerable target of SARS-CoV-2, and pituitary damage, whether direct or indirect, has been recognized as a determining factor of secondary hypothyroidism (functional or organic).^8^ As 3,5,3′-triiodothyronine (T3) and thyroxine (T4) are positively correlated with serum ACE levels, the ACE level could be used as a marker to investigate the action of peripheral thyroid hormones.^9^ A study by Rotondi et al. also proposed the thyroid as a potential target for SARS-CoV-2, as thyroid follicular cells encode the messenger RNA for ACE2 receptors.^10^

After entering the body, SARS-CoV-2 may cause numerous clinical symptoms, SARS and multisystem organ failure by causing both direct and indirect injury to the body. The direct effect is due to the cytotoxic effect of the virus on the target cell, and the indirect effect is caused by the aberrant immune inflammatory responses, which include cytokine, complement systems and coagulation.^6,7,9,11^ Innate and adaptive immune responses are regulated by thyroid hormones via genomic and nongenomic pathways.^12^ Cytokine production and release are triggered by T4 and T3; this results in a “cytokine storm”, which usually accompanies systemic viral infections.^13,14^

Furthermore, thyroid hormones can enhance the antiviral activity of interferon-γ, thus explaining why some immune system pathways, such as cytokine and T helper 1 cell hyperactivation, occur in response to virus infections in thyroid disorders.^12^ It is also worth noting that T4 is capable of activating human platelets, which lead to pathological clotting, which is a complication of virus infection.^15^ This article reviews up-to-date clinical information about the interactions between the thyroid gland and COVID-19, including thyroid pathology, thyroid function, drug interactions, cytotoxic effect and thyroid diseases.

## Methods

We conducted a literature search of MEDLINE, EMBASE and Google Scholar to identify articles reporting on thyroid dysfunction in patients diagnosed with COVID-19. Articles eligible for inclusion were observational cohort studies, case–control studies and randomized controlled trials, case reports and expert opinions published between 1 January 2019 and 15 March 2021. Search terms included “Sars-coV-2”, “coronavirus”, “COVID”, “subacute thyroiditis”, “De Quervain thyroiditis”, “subacute thyroiditis”, “non thyroidal illness syndrome”, “Hashimoto's thyroiditis”, “Graves' disease”, “hypothyroidism”, “coagulopathy”, “baricitinib”, “methimazole”, “REGN-COV2”, “remdesivir” and “tocilizumab”. No exclusions were made for language, disease severity or outcomes reported.

Two reviewers independently undertook a two-step selection, with studies screened via titles and abstracts followed by a full-text review. Data were extracted from the articles' text, tables and figures. Data were collected, analysed and discussed to answer the following clinical questions:

- Can COVID-19 have a negative impact on thyroid function?
- Is the COVID-19 prognosis worse with concomitant or previous thyroid disease?
- Could the clinical course of COVID-19 be altered by the treatment of thyroid diseases?
- Does COVID-19 treatment interfere with thyroid function?

This review discusses the relationship between COVID-19 and thyroid gland/thyroid-related diseases, including the direct cytological effects of the virus, the autoimmune-related effects, the hypothyroidism-related effects, the relationship to non-thyroidal illness syndrome (NTIS) and drug-related connections.

## Direct cytological effects

Patients infected with SARS-CoV-2 may present with various symptoms that may be related to an ongoing thyroid pathology or present variations in markers related to thyroid function.

According to a subgroup analysis of patients with COVID-19 conducted by Chen et al., levels of thyroid-stimulating hormone (TSH) and total T3 (TT3) depression positively correlated with the prognosis of the disease.^16^ This means that TSH and TT3 levels decline as the infection proceeds. Similar outcomes have been reported in two further studies.^17,18^ The symptoms observed in patients have different aetiologies. The primary hypothesis is the direct influence on the thyroid gland by SARS-CoV-2 causing euthyroid sick syndrome (ESS), resulting in decreased levels of serum T3 and/or T4 without increased secretion of TSH.^19^ In patients with COVID-19, ESS may be directly caused by the infection of thyroid cells with SARS-CoV-2. Although no article on the involvement of the thyroid gland in patients with COVID-19 has been published, varying degrees of damage to the thyroid gland were confirmed in patients infected with SARS back in 2007.^20^ We know that cytokine storms characterized by the uncontrolled and excessive release of inflammatory mediators and resulting in overwhelming systemic inflammation and even multiple organ dysfunction are very common in patients with COVID-19, especially in severe cases.^21^ Zou et al. reported that more than 25% of patients with COVID-19 were diagnosed with ESS.^22^ ESS is an independent risk factor for disease severity in patients with COVID-19. Patients with COVID-19 with ESS had stronger inflammatory responses and showed higher levels of C-reactive protein and erythrocyte sedimentation rate and a positive rate of procalcitonin.^22^ Therefore, direct virus effect and inflammation in COVID-19 seem to be responsible for ESS.

The other hypothesis explains the decreased serum TSH levels in patients infected by SARS-CoV-2 through a distorted hypothalamic– pituitary–thyroid axis.^23^ A critical function of serum TSH is the inverse proportional association with interleukin-6 (IL-6) levels, which identifies inflammatory response to SARS-CoV-2.^23^ If the IL-6 level is high in patients with COVID-19, there is a possibility of ESS, and low TSH and T3 levels are found. This may suggest that the prognosis may be poor. However, we need to know whether there is existing thyroid disease for us to confirm this interpretation. A patient's condition and serum TSH levels may also be explained by the relationship between serum TSH levels and IL-6 levels.

SARS-CoV-2-associated thyroid dysfunction can be caused through direct viral mechanisms. SARS-CoV-2 can disrupt many different tissues via indirect effects, such as inflammatory-mediated response, autoimmune-mediated response, coagulation and microthrombus. The initiation of proinflammatory responses, including several cytokines (e.g. IL-1β, IL-6, tumour necrosis factor-α and adaptive T-cell-mediated immune response), is triggered by an indirect mechanism of SARS-CoV-2.^24,25^ In addition, the post-viral inflammatory response can be responsible for thyroid dysfunction.^26^

## Autoimmunity-related effect

### Graves' disease

A highly common cause of thyrotoxicosis is the action of autoantibodies against TSH receptors, which mimic the action of TSH and activate the receptors, resulting in Graves' disease.^26^ Mateu-Salat et al. documented two cases of COVID-19-related Graves' disease: one with a history of Graves' disease documented as being in remission for over 30 years, and the other without any documented history of thyroid disease.^27^ It is known that Graves' disease is the most frequent hyperthyroidism aetiology.^28^ Thus, Mateu-Salat et al. suggest that physicians, especially endocrinologists, should be aware of a potential link between thyroid gland-related diseases involving autoimmune pathways and SARS-CoV-2, given the increasing number of publications documenting SARS-CoV-2 as acting as a trigger of latent or new-onset autoimmunity.^27^

### Subacute thyroiditis

Subacute thyroiditis (SAT; also known as de Quervain thyroiditis) is a self-limiting disorder consisting of three phases: painful swelling of the thyroid, hypothyroidism and euthyroidism. SAT is mostly caused by viral infections (e.g. mumps, measles, adenovirus, coxsackievirus, Epstein– Barr virus) but has also been reported following rickettsial and bacterial infections.^29^ To date, 25 cases of a post-infection, or presumed to be post-infection, SAT developing secondarily to SARS-CoV-2 infection have been reported.^30–47^ Most physicians who reported post-infection SAT emphasized the importance of patient monitoring and awarereness of possible SAT development. Hence, the effects of SARS-CoV-2 on thyroid tissue are not yet clear due to a lack of knowledge. Physicians should be aware of symptoms such as neck pain and persistent tachycardia, which might point to SARS-CoV-2-related SAT.^8^

## Hypothyroidism

Clinical studies exposed viral infection-related thyrotoxicosis cases represented as a post-viral inflammatory process followed by SARS-CoV-2 infection.^48–51^ As the clinical course of a thyrotoxic state proceeds, patients' thyroid hormone levels drop to the hypothalamic reference range.^52^ The evaluation of both viral and post-viral thyroiditis resulting in hypothyroidism may reveal SARS-CoV-2 infection, thus suggesting that physicians should be aware of a possible relationship between COVID-19 and hypothyroidism. This relationship may also be expressed by the effect of the virus on the hypothalamic–pituitary– thyroid axis.

Previous case studies have reported central hypocortisolism and low dehydroepiandrosterone sulfate, which, following evidence from autopsy reports documenting neuronal degeneration and oedema and investigations of the SARS-CoV-2 genome in the hypothalamus, may be considered signs of reversible hypophysitis or direct hypothalamic damage.^53^ The findings to date, together with the fact that ACE2 is highly expressed in the pituitary gland and hypothalamus compared with other central nervous system-related tissues, suggest that the relationship between SARS-CoV-2 and hypothyroidism requires further investigation.^53^

## Non-thyroidal illness syndrome

NTIS, also known as ESS, is defined as decreased serum T3 and/or T4 levels without any essential increase in TSH secretion. NTIS manifests during critical conditions, such as infections, trauma, myocardial infarction and malignancy, or in the fasting state of a healthy individual. As in the fasting state, such conditions may lead the body into an energyconserving state as an adaptive reaction to protect the body from stress factors. Studies have suggested that the severity of NTIS is inversely correlated with the likelihood of NTIS-related pathology.^16,54^ In their study, Chen et al. also suggested a similar relation between the severity of patients with COVID-19 and NTIS-dependent serum T3/T4 hormone levels.^16^ The study by Lui et al. revealed an inversely proportional erythrocyte sedimentation rate and an FT3/FT4 ratio, which might indicate the effect of systemic inflammation on deiodinase activity.^54^

## Coagulopathy

Coagulation is a complex response of the body to damaged endothelial cells and involves many inflammatory cytokines and chemokines, such as tumour necrosis factor-α, IL-1, IL-6 and IL-8.^55^ This response can lead to coagulopathy when another factor or factors cause disruption in elements responsible for the physiological coagulation process. Previous studies have suggested that cytokines related to coagulation are also involved in the inflammatory process of SARS-CoV-2; furthermore, coagulation disorders caused by SARS-CoV-2 can lead to a serious hypercoagulable state in critically ill patients.^55^

## Drugs

In the earlier phases of the COVID-19 pandemic, physicians struggled to understand the disease and treat patients, especially those with a severe illness. The difficulties with treatment were related to COVID-19 disease traits with which physicians were unfamiliar. Since the pandemic, research conducted by physicians worldwide has aimed to enlighten the medical community and establish efficient treatment approaches and effective drugs; however, collecting conclusive evidence can be slow due to a lack of reliable data to generate decisive treatment algorithms and to the different variants of SARS-CoV-2 circulating. For the past 2 years, most physicians have considered COVID-19 the top research priority to gather meaningful data from which to construct treatment algorithms, including the use of drugs for empirical treatment.

The COVID-19 Treatment Guidelines Panel of the National Institutes of Health (NIH) issued guidance on COVID-19, including suggestions and exclusions of different drugs and drug types, as well as therapeutic procedures for managing COVID-19.^56^ Antiviral agents with different mechanisms of action were initially regarded as the primary treatments for patients with SARS-CoV-2 infection. Today, a range of agents are recommended: antiviral agents acting as nucleotide analogues (remdesivir [Veklury^®^, Gilead Sciences, Foster City, CA, USA]), which are intracellularly metabolized to an analogue of adenosine triphosphate that inhibits viral RNA polymerases; protease inhibitors (lopinavir [Kaletra^®^, AbbVie, North Chicago, IL, USA); antiviral drugs that target inflammation via a different mechanism, such as glucocorticoids (dexamethasone [Pfizer CentreOne^®^, New York, NY, USA]), which modulate inflammation-mediated lung injury and thereby reduce the progression to respiratory failure; kinase inhibitors (baricitinib [Olumiant^®^, Eli Lilly and Company, Indianapolis, Ind., USA]); and immunosuppressive drugs (tocilizumab [Actemra^®^, F. Hoffmann-La Roche AG, Basal, Switzerland]) acting against immunomodulatory proteins, such as IL-6. Drugs have also been recommended for preventing complications and comorbidities, such as coagulation disorders, which may be prevented with heparinoids, and shock, which may be treated with norepinephrine. As insufficient data are available on the interactions between thyroid function and the various COVID-19 pharmacological treatment options, conclusive statements cannot be drawn about their role in patients with thyroid disease.^56^

### Remdesivir

Remdesivir is an adenosine triphosphate analogue with a broad antiviral activity that first appeared in the literature in 2016 as a potential treatment for Ebola. The drug uses an RNA-dependent RNA polymerase enzyme mechanism acting on the Filoviridae, Paramyxoviridae, Pneumoviridae and Coronaviridae families.^57^ Physicians and researchers decided to try using remdesivir to treat patients with COVID-19 based on theoretical assumptions alone, as there was no prior knowledge of the virus and its effect on patients infected with SARS-CoV-2. Following clinical trials, research studies and data evaluation of the drug, remdesivir was granted a US Food and Drug Administration (FDA) Emergency Use Authorization (EUA) on 1 May 2020.^58^ However, remdesivir may cause hepatotoxicity in certain situations.^59^

In terms of the interactions between thyroid-related dysfunction and COVID-19, the concomitant use of thionamides and remdesivir is not advised due to the risk of hepatotoxicity unless both drugs are critical to the patient; in such cases, decisions to administer both drugs should be taken under supervision.^60^

### Baricitinib

Baricitinib is a proinflammatory pathway inhibitor acting as a selective and reversible Janus kinase 1 (JAK1) and 2 (JAK2) inhibitor. Baricitinib is usually used for treating rheumatoid arthritis to prevent inflammatory damage to the joints.^61^ Baricitinib was approved in February 2017 by the EU as a therapeutic option for rheumatoid arthritis.^61^

The effects of baricitinib on JAK1 and JAK2 enzymes were also observed in patients with COVID-19 suffering from moderate-to-severe inflammatory responses.^56^ Baricitinib has been approved for use by the FDA EUA. NIH treatment guidelines recommend that the use and dosage of baricitinib in patients with COVID-19 be determined based on the estimated glomerular filtration rate.^56^

The use of baricitinib might cause adverse events in patients with rheumatoid arthritis or COVID-19. However, there are no reliable data on any interactions between baricitinib and thyroid drugs used for both hypothyroiditic and hyperthyroiditic states. Furthermore, the use of baricitinib has no notable effect on patients suffering from thyroid-related complications besides its adverse effects shown by prior research.^62,63^

Baricitinib, administered for atopic dermatitis, did not induce adverse thyroid events;^62,63^ however, it remains unclear whether the possible onset of thyroid autoimmunity could be attributable to an underlying autoimmune condition rather than the consequence of an adverse event.^63^

Baricitinib plus remdesivir was superior to remdesivir alone in reducing recovery time and accelerating improvement in clinical status among patients with COVID-19, notably those receiving high-flow oxygen or noninvasive ventilation.^62^ The combination was associated with fewer serious adverse events.^62^

### Tocilizumab

Tocilizumab is another drug used for rheumatological disorders, such as giant cell arteritis, rheumatoid arthritis and systemic juvenile idiopathic arthritis. Tocilizumab acts as an antibody IL-6 receptor inhibitor, resulting in a reduction in undesired inflammatory and autoimmune activities in IL-6, which may negatively impact on patients suffering from SARSCoV-2.^64^ The progression of COVID-19 involves inflammatory processes related to the IL-6 mechanism.^64^ As an IL-6 receptor inhibitor, tocilizumab appeared to be a beneficial drug to use in some COVID-19 cases and was suggested by the NIH COVID-19 Treatment Guidelines Panel.^51^ In the same guideline, another IL-6 inhibitor, sarilumab, is also suggested if tocilizumab is not available.^56^

Autoimmune inflammatory responses triggering IL-6 mechanisms might be prevented by tocilizumab; however, the interaction of the drug with the human autoimmune system may result in some adverse effects. By including thyroid-related diseases into the equation, some adverse effects of tocilizumab might be confused with the possible onset of thyroid autoimmunity.^65^

### Dexamethasone

Dexamethasone is a glucocorticoid used to treat several different disorders, including endocrine, rheumatic, collagen, dermatological, allergic, ophthalmic, gastrointestinal, respiratory, haematological, neoplastic and oedematous disorders, by inhibiting proinflammatory signals and promoting anti-inflammatory signals.^66,67^ Dexamethasone has a relatively long history of therapeutic usage by physicians and was granted FDA approval on 30 October 1958.^68^ Recently, the NIH Treatment Guidelines Panel and the WHO guidelines on drugs for COVID-19 have included the use of dexamethasone for managing COVID-19. Cumulative data collected on patients needing supplemental oxygen showed a reduction in the 28-day mortality rate to 29.3% from 41.4%.^69^ However, dexamethasone is contraindicated in patients with several thyroid-related diseases. Research conducted by Vigneri et al. showed that short-term dexamethasone administration decreased basal T3 serum concentration, while the T3 incremental response to TSH was not altered.^70^ Considering the importance of glucocorticoid use in severe COVID-19 cases, patients with prior thyroid-related diagnoses should be treated carefully given the adverse systemic effects of dexamethasone.

### Regn-Cov2

REGN-COV2 (Regeneron Pharmaceuticals, Inc., Tarrytown, NY, USA; Roche, Basel, Switzerland) is a monoclonal antibody combination consisting of casirivimab and imdevimab. Both monoclonal antibodies operate to neutralize the spike protein of SARS-CoV-2. The FDA authorized the emergency approval of REGEN-COV2 to treat mild-to-moderate COVID-19 in patients aged 12 years and older.^71^ The NIH COVID-19 Treatment Guidelines Panel recommend REGN-COV2 for non-hospitalized patients. As both drugs are not officially approved by the FDA (except for emergency approval), it is not possible to suggest or disprove the use of REGN-COV2 with any thyroid-associated disease.^56^

### Drug use in complications

COVID-19 and its effects on other comorbidities or indirect mechanisms are still under investigation. Throughout the pandemic, physicians have experimented with different management approaches for different complications, such as coagulopathy. The NIH COVID-19 Treatment Guidelines Panel recommends the use of heparinoids in hospitalized patients with COVID-19 for prophylactic purposes.^51^ Recent studies and data suggest that physicians should be aware that low-molecular-weight heparin in patients with thyroid-related diseases may disrupt thyroid function and affect laboratory findings.^72^ Heparin enhances endothelial lipoprotein lipase activation and increases serum non-esterified fatty acid (NEFA) concentration. After NEFA concentrations surpass the serum-binding capacity threshold, NEFA acts as a direct competitor of T4 and T3 binding sites located on thyroxine-binding globulin.^73^ Therefore, hospitalized patients with SARS-CoV-2 infection with any T3 and T4 hormone need measurement should be evaluated carefully if the patient is receiving prophylactic heparinoid therapy.

Whether related to SARS-CoV-2 infection or not, complications must be evaluated and treated carefully due to the risk of unexpected response to the infection. The NIH COVID-19 Treatment Guidelines Panel recommends drugs to treat conditions such as shock, acute kidney injury and co-infections with influenza/HIV; these recommendations follow the respective original guidelines published for these conditions.^56^ Physicians should treat patients with thyroid-related disorders very carefully if they also require concomitant medications.

## Conclusion

Routine thyroid function monitoring is not needed for patients with COVID-19 infection without any known thyroid dysfunction, routine thyroid function monitoring is not needed. However, for COVID-19 patients with previous or ongoing thyroid dysfunction or clinical presentations of hypothyroidism/hyperthyroidism, monitoring of their thyroid function is advised.
